# College and Hospital Notes

**Published:** 1901-07

**Authors:** 


### COLLEGE AND HOSPITAL NOTES.

---

THE Albany Hospital has arranged a series of clinical days for the months of July and August, to which practitioners of medicine are invited. They will occur once a week and will consist of clinical lectures and demonstrations in general medicine, major and minor surgery, gynecology, pathology, clinical microscopy and the specialties. An entire day, Tuesday of each week, will be thus occupied and an effort will be made to present the most valuable of the recent advances in medicine with illustrative cases. The work will be thoroughly practical and helpful, and is especially designed for those who are desirous of keeping in touch with medical progress as shown in a large, modern, and well-equipped hospital.

The Albany Club extends to the non-resident physicians in attendance the courtesies of the club house. These privileges are made available through a card, which may be obtained from the secretary of the course. Physicians who contemplate attending the clinical days are requested to register with the secretary of the course, in order that they may receive on each successive Monday a definite and detailed program of the succeeding clinical day.

The provisional program may be at times modified, in order that rare or unusual cases may be presented. No charge whatever will be made for any of the clinics or lectures. Physicians who desire further information can obtain full details by addressing Dr. Edgar A. Vander Veer, Secretary of the Course, 28 Eagle Street, Albany, N.Y.

---

University of Buffalo—Medical Department—A Pan-American Bureau of Information for use of alumni exclusively has been established by the faculty and Alumni Association. Office, 24 High Street—near Main Street—in the library of University building.

It costs nothing, the object being to secure comfort, to save annoyance to the visitors. Every U. of B. man and woman is anxious to show you courtesy, so please wear your U. B. pin conspicuously.

Accommodations secured in advance, if desired.

Hotels, rooms and restaurants on the list all have the personal endorsement of some well-known Buffalo alumnus.

Mail, telegrams, and packages sent in care of this bureau will get prompt and proper attention.

The register will give the date of arrival, stopping place, time and route of departure and future address, if desired.

Hours—Bureau open from 8 a.m. to 6 p.m. At other hours mail, etc., can be obtained at the janitor's, No. 27 Goodrich Street —next north of High—in rear of College building. Telephone.

A room for lounging, reading and writing is in operation.

Car to all gates of the Exposition pass the corner near by.

To get there—take street cars marked Main at Main Street, which are going North and stop at High Street. The University building is next the corner.

---

The Gowanda State Hospital will be enlarged by two wings that will be added to the present building or set of buildings. Each wing will cost at least $75,000, making the total expenditure $150,000. Provision for the money was made in past appropriation measures by the legislature, so there is no likelihood of failure or delay.

---

The Cornell University Medical College, according to the New York *Tribune*, has recently acquired the library of the late Dr. Felix Victor Birch Hirschfield, who was professor of pathology and pathological anatomy at the University of Leipsic and director of the Leipsic Pathological Institute. This library which contains about 5,000 volumes, is said to be one of the most valuable pathological libraries in existence. It is a collection of the periodical literature of Germany, France and England on pathological subjects for more than fifty years, and contains detailed descriptions of the many discoveries made in that time.

The purchase was made by Prof. James Ewing in the name of the Loomis Laboratory. About $10,000 was paid for the books.

